# Mindfulness and Rumination: Uncovering Stress Pathways in Caregivers of Persons Living with Dementia

**DOI:** 10.1093/geroni/igaf122.2655

**Published:** 2025-12-31

**Authors:** Jyoti Savla, Marrium Mansoor, Lauren Hagemann, Katherine Lucci, Mamta Sapra

**Affiliations:** Dept. of Human Development and Family Science, Blacksburg, Virginia, United States; Virginia Tech, Blacksburg, Virginia, United States; Salem VA Healthcare System, Salem, Virginia, United States; Salem VA Healthcare System, Salem, Virginia, United States; Salem VA Healthcare System, Salem, Virginia, United States

## Abstract

Mindfulness techniques, which encourage focusing on the present moment, have shown promise in reducing stress. However, the mechanisms through which mindfulness-based interventions alleviate stress in family caregivers, particularly those caring for persons living with dementia, remain less understood. This study aimed to determine whether a mindfulness-enhanced psychoeducational intervention could lower stress among caregivers of persons living with dementia by reducing rumination – a repetitive pattern of negative thinking. Data were drawn from a randomized controlled trial involving 142 family caregivers of veterans living with dementia (Mage = 67.2, SD = 9.8; 85% women; 82% White; 70% spouse caregivers; 90% co-residing). Participants were randomly assigned to either a standard REACH-VA multi-component skill-building intervention (n = 66) or a mindfulness-enhanced skill-building intervention, named PAACC (n = 67). Data were collected at baseline and at the end of the study. Rumination predicted higher depressive symptoms (using the Patient Health Questionnaire) and perceived stress (using the Perceived Stress Scale), but not caregiver burden (using the Zarit Burden Interview). Additionally, moderated mediation analysis showed that the relationship between anxiety (on the Generalized Anxiety Disorder Screener) and rumination (Rumination Reflection Questionnaire) was significantly stronger in the mindfulness-enhanced intervention group. These findings suggest that the mindfulness-enhanced intervention may be particularly beneficial for caregivers with higher anxiety levels by interrupting the cycle of negative, repetitive thinking, thereby reducing emotional stress even if caregiving burden remains unchanged. Incorporating mindfulness techniques may, therefore, provide meaningful benefits for caregivers by specifically targeting anxiety-driven rumination and improving emotional well-being.

